# Community Engagement Alliance (CEAL) Against COVID-19 Disparities: Academic-community partnership to support workforce capacity building among Arizona community health workers

**DOI:** 10.3389/fpubh.2023.1072808

**Published:** 2023-02-02

**Authors:** Dulce J. Jiménez, Omar Gomez, Ruby Meraz, Amanda M. Pollitt, Linnea Evans, Naomi Lee, Matt Ignacio, Katherine Garcia, Richard Redondo, Floribella Redondo, Heather J. Williamson, Sabrina Oesterle, Sairam Parthasarathy, Samantha Sabo

**Affiliations:** ^1^Center for Health Equity Research, Northern Arizona University, Flagstaff, AZ, United States; ^2^Arizona Community Health Workers Association, Douglas, AZ, United States; ^3^Department of Chemistry and Biochemistry, Northern Arizona University, Flagstaff, AZ, United States; ^4^Southwest Interdisciplinary Research Center, School of Social Work, Arizona State University, Phoenix, AZ, United States; ^5^Division of Pulmonary, Allergy, Critical Care and Sleep Medicine, Department of Medicine, University of Arizona, Tucson, AZ, United States

**Keywords:** community health worker (CHW), community health representatives, COVID-19 pandemic, academic-community partnership, mixed methods, Latinx/Hispanic, American Indian/Indigenous

## Abstract

The COVID-19 pandemic has both highlighted and worsened existing health inequities among communities of color and structurally vulnerable populations. Community Health Workers, inclusive of Community Health Representatives (CHW/Rs) have entered the spotlight as essential to COVID-19 prevention and control. To learn about community experiences and perspectives related to COVID-19 and inform CHW/R workforce capacity building efforts, a series of focus groups were conducted with CHW/Rs throughout Arizona at two time points in 2021. Throughout the data collection and analysis process, researchers and community partners engaged in ongoing and open dialogue about what CHW/Rs on the ground were reporting as priority community concerns, needs, and challenges. Thus, CHW/Rs informed the development of culturally and linguistically relevant health education messages, materials, and training for CHW/Rs. In this community case study, we detail the efforts of partnership between a statewide CHW professional association and an academic research team that facilitated rapid decision-making and knowledge sharing to create community-grounded tools and resources supportive of CHW/R workforce capacity building in the context of the COVID-19 pandemic.

## 1. Introduction

The COVID-19 pandemic has both highlighted and worsened existing health inequities among communities of color and structurally vulnerable populations (1–3). Various studies have documented racial, ethnic, and socioeconomic disparities in COVID-19 incidence, hospitalizations, and mortality, with African American, Latinx/Hispanic, and American Indian/Alaska Native populations bearing the greatest burden (2–6).

In tandem with the COVID-19 pandemic, the world has also witnessed an infodemic or an excess of rapidly spreading information in both the physical and digital global environments, including the spread of false news and misinformation that have undermined public health efforts in the fight against COVID-19 (7–10). Community health workers (CHWs) – inclusive of tribally employed community health representatives (CHRs) and promotores de salud among other titles – have entered the spotlight as powerful assets in COVID-19 prevention and control, including addressing misinformation (11, 12). CHWs are frontline public health workers who are trusted members of the communities they serve and as such, have a unique understanding, sharing the culture, language, and lived experiences of the clients they serve (13, 14). As valuable members of public health and care teams who are effective in reducing health disparities and improving health outcomes, CHWs play a vital role in addressing medical and social determinants of health (SDoH) among underserved populations (14). As experts who represent and advocate for the communities they serve, CHWs may also help ensure the social validation of goals, procedures, and effects for public health interventions, for example by aligning research goals with community concerns, assessing the acceptability and relevance of study methods, and evaluating the social significance of outcomes (15). Globally, CHWs have been pivotal in pandemic responses, especially in low and middle income countries (12, 16) and are well-positioned to play essential roles in community-based COVID-19 responses now and in the future (11).

CHWs serve as a bridge between community members and fragmented systems of care, and can support efforts to ease fear and correct false information in disadvantaged communities by leveraging their cultural connectedness and shared lived experiences to offer trusted advice and education (17–19). Examples of how CHWs have mobilized during the pandemic include connecting clients to basic services such as food pantries and food distribution sites, rent assistance, primary care providers, and mental health resources; navigating health systems, complex unemployment filing systems, and resources for undocumented immigrants; facilitating delivery of medications; creating and disseminating culturally and linguistically relevant health education materials; and providing social support to isolated older adults *via* phone (17, 18). CHWs have the expertise, connection, and credibility needed to address the overwhelming misinformation around COVID-19 and its potential to have devastating impacts in underserved communities, including rural areas (18, 20). However, the ever-evolving nature of COVID-19 and related public health guidance has made it difficult for both communities and health professionals, including CHWs, to stay updated on the most current and accurate information, highlighting the need for ongoing education efforts.

## 2. Context

Since the beginning of the pandemic, Arizona has been one of the hardest-hit states in the US, continuously ranked among the highest in case and death rates, at times having the highest rate of new cases in the country (21) and even in the world twice (22). With close to 2 million all time COVID-19 cases, nearly 28,000 deaths (23), and a population of almost 7.3 million (24) policies at the Arizona state level have been insufficient to effectively prevent and control the spread of COVID-19. In light of regular spikes in COVID-19 cases since 2020, Arizona's Governor Doug Ducey routinely denied science based COVID-19 prevention and control measures, including declining to institute a statewide mask mandate, allowing businesses to remain open, and letting school districts make their own decisions about operations (25).

In September of 2020, the National Institutes of Health (NIH) funded the Community Engagement Alliance (CEAL) Against COVID-19 Disparities in 11 of the hardest hit states, including Arizona, to conduct outreach and engagement efforts in ethnic and racial minoritized communities disproportionately affected by the COVID-19 pandemic (26). The Arizona CEAL Consortium (AZ-CEAL) is a collaboration of Northern Arizona University (NAU), University of Arizona (UA), Arizona State University (ASU), Mayo Clinic, and the Arizona Community Health Worker Association (AzCHOW). In partnership with members and leaders of African American, Hispanic/Latinx, and American Indian communities, the Arizona CEAL aims to provide trustworthy information through active community engagement and outreach to the people hardest-hit by the COVID-19 pandemic, with the goal of building long-lasting partnerships as well as improving diversity and inclusion in our research response to COVID-19. Specifically, the purpose of AZ-CEAL is to:

(1) Conduct community-engaged research and outreach to assess awareness, experiences, concerns, attitudes, needs, knowledge, and misconceptions regarding COVID-19 testing, prevention, research participation, vaccination uptake, and medical mistrust.
(2) Develop culturally-appropriate dissemination materials and strategies designed to educate about COVID-19 infection, transmission prevention, testing, and vaccination; decrease misinformation; and increase medical trust.
(3) Implement and evaluate the dissemination of materials and educational strategies on enhancing awareness, trust, willingness, ability, self-efficacy, and participation, of underserved communities in advancing the prevention and treatment of COVID-19.

This paper focuses on NAU's Center for Health Equity Research (CHER) equitable partnership with AzCHOW, an Arizona organization of community-based advocates that has been advocating for the work of CHW/Rs in the state since 2001. AzCHOW builds CHW/R capacity across disciplines to address CHW workforce policy and sustainability issues while serving Arizona's underserved and at-risk populations. By way of resource sharing, partnership development, education, outreach, health promotion, and disease prevention strategies, AzCHOW works toward improving the health of Arizona residents (27). NAU and AzCHOW collaborated to develop and disseminate accurate, up-to-date COVID-19 information for CHWs to take back to their communities.

Previous work highlights the importance of engaging CHW professional associations and CHWs in the research process, including substantive pieces such as designing the study, collecting, analyzing, and interpreting data, and disseminating findings (28, 29). Following best practices in community-engaged research, NAU and AzCHOW partnered equally and intentionally involved CHWs throughout the research process and resulting CHW capacity building training. This collaboration between researchers, a CHW professional association, and CHWs facilitated targeted and methodologically sound research by ensuring effective and relevant study design, recruitment and participation, and action following research findings (28, 29).

In Arizona, the CHW workforce is estimated at 1500–2000 CHWs employed in county and tribal health departments, health centers, schools, churches, and not-for-profits. Furthermore, the CHR workforce, comprised of tribally employed CHWs, is the oldest and only federally funded CHW workforce in the United States, consisting of a highly trained, well established standardized workforce serving the medical and social needs of American Indian communities (30). Since both CHWs and tribally employed CHRs were engaged in this study, we will use the title CHW/R.

As frontline responders who are also members of the communities they serve, CHW/Rs have a unique understanding of their community's experiences during the COVID-19 pandemic. To learn about community experiences and perspectives related to COVID-19 and inform CHW/R workforce capacity building efforts lead by AzCHOW, a series of focus groups were conducted with CHW/Rs throughout the state. Focus group conversations with CHW/Rs happened at two time points in 2021 and were aimed at exploring CHW/R client experiences and perspectives related to COVID-19 and CHW/R experiences, strategies to overcome challenges, and professional training priorities during the pandemic. The information shared during focus groups was used to develop and adapt COVID-19 educational materials and health education messages, and to identify knowledge gaps and training priorities for CHW/Rs. In this community case study, we detail the efforts of partnership between a statewide professional association (AzCHOW) and an academic research team (NAU) that facilitated timely data collection and analysis for rapid decision-making and action to support the Arizona CHW workforce during the COVID-19 pandemic.

## 3. Key programmatic elements

### 3.1. Survey and focus groups

We employed a highly participatory research approach that included AzCHOW staff and CHW/Rs in all phases of the work, from study design to data collection and dissemination. To learn about CHW/R client challenges, needs, and misinformation related to COVID-19 as well as CHW/R experiences, strategies to address COVID-19 challenges, and their training priorities, we conducted focus groups with volunteer and employed CHW/Rs at two distinct time points during the COVID-19 pandemic. The focus group guide was designed for semi-structured discussion and included questions such as, “What types of misinformation or myths have your clients shared with you about COVID-19 that you know or believe is not true and how are you correcting these myths?” and “What are the priority training topics and tools that you want and need to support your clients?” Qualitative research methods were adapted over time to meet the rapidly shifting context of the pandemic and address the urgent education and communication needs of the CHW/R workforce and their clients. Prior to conducting phase 1 and 2 focus groups, research staff conducted two pilot focus groups in English and Spanish in December 2020 to test the focus group guide tool with CHW/Rs.

#### 3.1.1. Recruitment

CHW/Rs were recruited using purposive sampling primarily through AzCHOW's email listserv, which includes approximately 460 email addresses for CHW/Rs, CHW/R supervisors, and partners. Recruitment also happened through word of mouth from CHW/R colleagues and research staff. A total of *N* = 54 participants took part in focus group sessions during the two phases. Of the 54 CHW/Rs, 8 participated in both phase 1 and phase 2 focus groups, leading to a total of *N* = 46 unique Arizona CHW/Rs. To reach tribally employed CHW/Rs serving American Indian populations living on tribal lands, we obtained human subjects research approval from three tribes and recruited CHW/Rs employed by the program using flyers shared *via* the CHR supervisor. Tribes will remain anonymous as per tribal approval agreements. All procedures for this study were approved by the University of Arizona Institutional Review Board (Protocol # 2011244240).

#### 3.1.2. Data collection and analysis

Before participating in the focus groups, CHW/Rs completed a brief online survey *via* Qualtrics. The online survey assessed CHW/R demographics (i.e., race/ethnicity, age, gender, education, and employment), COVID-19 prevention behavior (i.e., frequency of mask use, hand washing, and social distancing), likelihood to get the COVID-19 vaccine, and trusted sources of COVID-19 information.

##### 3.1.2.1. Phase 1

The first set of focus groups (*N* = 10 sessions) were conducted between January–March 2021. At the beginning of February 2021, Arizona had 762,593 total COVID cases (31), 13,124 total deaths (31) and had administered 883,808 doses of the COVID-19 vaccine with only a fraction of all eligible Arizonans fully vaccinated (2.2%, *N* = 147,595 for population age 5 years or older) (32).

This initial phase consisted of 10 focus groups, where 30 Hispanic/Latinx CHWs participated in 7 focus groups and 11 American Indian CHRs participated in 3 additional focus groups (*N* = 41 total participants employed across the state of Arizona). Focus groups were conducted virtually through Zoom by bilingual, bicultural research staff representative of Latinx and Indigenous lived experiences, in the English and Spanish language. Conversations lasted 90 min and were audio recorded and transcribed verbatim by research staff. Audio and transcript files were then reviewed, and a code book was developed from the data. Four research staff independently coded the transcripts in ATLAS.ti 8 and identified common themes for each focus groups conversation topic through a process of consensus.

##### 3.1.2.2. Phase 2

The second set of focus groups (*N* = 4 sessions) were conducted in August 2021. At the beginning of August 2021, Arizona had 929,541 total COVID cases (31), 18,251 total deaths (31), and had administered 7,636,771 doses of the COVID-19 vaccines with just over half of all eligible Arizonans fully vaccinated (50.9%, *N* = 3,438,112 for population age 5 years or older) (32).

This second phase consisted of 4 focus groups, where 8 Hispanic/Latinx CHWs participated for a second time in 2 focus groups and 5 new American Indian CHRs participated in 2 additional focus groups (*N* = 13 total participants employed across the state of Arizona). At this phase, and to allow for rapid planning and action among decision-makers involved in the COVID-19 response, a rapid assessment procedure (RAP) was used to collect and analyze phase 2 focus group data. The RAP tool was adapted based on real-time evaluation, iterative methodology (33), and rapid appraisal methods (34) to guide the focus group data collection, analysis, and output to enhance decision-making. The tool comprised a field annotation template of pre-defined constructs developed using the focus group conversation guide questions. Detailed field notes were made by three to four trained research staff during the focus groups using the template to generate focused results. Field annotation notes were summarized, and salient themes were identified through consensus among the entire NAU-AzCHOW team.

All phases of analysis and interpretation were conducted in collaboration with the AzCHOW team representative of CHWs and CHRs.

#### 3.1.3. CHW/R participant demographics

Table 1 summarizes demographic characteristics and Table 2 describes likelihood to get vaccinated for COVID-19, COVID-19 prevention behaviors, and trusted sources of COVID-19 information for the 46 unique CHW/Rs that participated in the focus groups across the two phases.

**Table 1:** Community health worker/representative (*N* = 46) demographics – combined phases 1 and 2.

**Variable**	**Percent (Frequency)**
**Age (*****N** =* **28)**	
25–40	50.0% (14)
41–50	21.4% (6)
50+	28.6% (8)
**Race/Ethnicity**	
Hispanic, any race including American Indian	73.9% (34)
Non-Hispanic American Indian	23.9% (11)
Non-Hispanic White	2.2% (1)
**Gender Identity**	
Woman	89.1% (41)
Man	10.9% (5)
**Education**	
High school or GED	43.5% (20)
Associate's or technical degree	41.3% (19)
Bachelor's degree	8.7% (4)
Prefer not to answer	6.5% (3)

**Table 2:** Community health worker/representative (*N* = 46) vaccine likelihood, COVID-19 prevention behaviors, and trusted sources of information – combined phases 1 and 2.

**Variable**		**Percent (Frequency)**		
**Likelihood of getting a COVID-19 vaccine**				
Not at all	6.5% (3)				
3	2.2% (1)				
4	6.5% (3)				
5	2.2% (1)				
6	2.2% (1)				
Very likely	80.4% (37)				
**COVID-19 prevention behaviors when in public in the past 7 days**	**Never**	**Some of the time**	**Very often**	**All of the time**
Hand washing	–	–	10.9% (5)	89.1% (41)
Mask use	2.2% (1)	2.2% (1)	8.7% (4)	87.0% (40)
Social distancing	–	4.4% (2)	39.1% (18)	56.5% (26)
**How much do you trust each of these sources to provide correct information about COVID-19?**	**Not at all**	**A little**	**A great deal**	**Don't know**
Your doctor or health care provider	2.2% (1)	23.9% (11)	73.9% (34)	–
The U.S. Coronavirus Task Force	2.2% (1)	50.0% (23)	43.5% (20)	2.2% (1)
Your faith leader	15.2% (7)	30.4% (14)	34.8% (16)	19.6% (9)
People you go to work or class with or other people you know	2.2% (1)	60.9% (28)	23.9% (11)	13.0% (6)
Arizona State Government	8.7% (4)	60.9% (28)	23.9% (11)	6.5% (3)
The U.S. government	15.2% (7)	60.9% (28)	19.6% (9)	4.4% (2)
Your close friends and members of your family	8.7% (4)	60.9% (28)	19.6% (9)	10.9% (5)
Local Tribal Government	8.7% (4)	50.0% (23)	19.6% (9)	21.7% (10)
Neighboring Tribal Government	10.9% (5)	47.8% (22)	15.2% (7)	26.1% (12)
News on the radio, TV, online, or in newspapers	21.7% (10)	58.7% (27)	8.7% (4)	10.9% (5)
Your contacts on social media	54.4% (25)	37.0% (17)	–	8.7% (4)

Across the two phases, CHW/R participants predominantly identified as women (89.1%, *N* = 41), having completed a minimum of a high school or GED education (93.5%, *N* = 43), and a balanced range in age with half being between 25 and 40 years old and the other half 41 and older. About three-quarters of CHW/Rs were Hispanic of any race (73.9%, *N* = 34) including three (6.5%) who were Hispanic American Indian CHW/Rs, and the remainder identified as non-Hispanic American Indian (23.9%, *N* = 11) and one (2.2%) non-Hispanic white participant. See Table 1.

CHW/R participants in both phases largely engaged in COVID-19 primary prevention behaviors, with 95.7% (*N* = 44) reporting wearing masks very often or all of the time, 100% (*N* = 46) washing their hands very often or all of the time (*N* = 46, 100%), and 100% (*N* = 46) practicing social distancing at least sometimes. The majority of CHW/R participants reported that they were very likely to get the COVID-19 vaccine (80.4%, *N* = 37) months before the vaccines were widely available. The top three sources CHW/Rs trusted the most to provide correct information about COVID-19 were their own doctors or health care providers (73.9%, *N* = 34), the U.S. Coronavirus Task Force (43.5%, *N* = 20), and their faith leaders (34.8%, *N* = 16). The least trusted source was their own contacts on social media, with 54.4% (*N* = 25) of CHW/Rs reporting trusting them “not at all”. Approximately half to two thirds of CHW/Rs reported trusting the remaining sources “a little” (i.e., close friends or family; people they work with, classmates, or others they know; news on the radio, TV, online, or in newspapers; US government; Arizona state government; local tribal government; and neighboring tribal government) see Table 2.

#### 3.1.4. CHW/R-identified priority training topics

CHW/R participants identified priority training areas based on their client's challenges and needs. The most salient priority area among both CHWs serving Latinx communities and CHRs serving AI communities was COVID-19 vaccines, including topics such as vaccine contents, safety and side effects, efficacy, and benefits. COVID-19 information was rapidly evolving, sometimes changing from 1 day to the next, and CHW/Rs needed accurate and up to date information on the COVID-19 vaccines to support them answer their client's questions, respond to their concerns, address misinformation, and help them make informed decisions about vaccination.

“Many may ask “what's the difference between Pfizer and Moderna?” What is the difference? […] just give me the nitty gritty. The current, what does it contain, what are the side effects, if any? Give me the worst case scenario and then tell me the positives, so I can promote that it is a good thing.”

“For me, it would be just having a basic understanding on how the vaccine works, because I had somebody asked me how effective it was, and I wasn't sure how to answer that because there's two vaccines right now that are out and they're different. I just don't know the difference between them. So, for me, it would be knowing exactly how it works and being able to relay that in a way.”

Another priority training area for CHW/R participants was mental health, including topics of anxiety and depression, isolation, grief, and loss. Again, both Latinx-serving CHWs and AI-serving CHRs expressed the importance of mental health resources to support their clients as well as their own mental wellbeing during the pandemic. During the COVID-19 pandemic, CHW/Rs faced mental health challenges in their work and the highlighted the need for appropriate self-care training for themselves and their clients. Due to the nature of their work, CHW/Rs were exposed to health risks related to COVID-19 as well as mental health risks in navigating emotionally difficult situations with their clients. The emotional toll of working with grieving clients who lost family and friends was not new, but the pandemic highlighted these experiences and exposed the need for self-care techniques and mental health resources.

“PTSD could be something from COVID just especially with people who lost loved ones, grieving processes, that'd be something, a training that would be good to work with just families that were affected with COVID, losing loved ones and just being scared of the world or going back out in the world after all this passes.”

“Preparing ourselves a little because of everything we were seeing. Including our own mental health because if our mental health is not well, how are you going to help other people? [...] We took the first 6 session training on different topics, but all based on mental health. I think that's very important, mental health training, so we don't try to bear all the burden ourselves [...] well it is part of our job to help search for resources, but if someone is not well, I don't think we could do a good job. So, I think a mental health training program would be good.”

In addition to mental health topics, CHW/R participants described the importance of maintaining physical health and wellbeing during the pandemic. CHW/Rs reported challenges in their clients managing chronic diseases, especially during periods of isolation. To support their clients, CHW/Rs recommended incorporating nutrition, physical activity, and chronic disease management topics into COVID-19 prevention training to further develop their own knowledge and skills in these areas. Thinking of long-term solutions, CHW/Rs suggested that training in this area should prepare CHWs and clients to adapt to new lifestyles during and after quarantine to manage and reduce the risk of chronic diseases.

“Most of our patients have health issues like diabetes and high blood pressure and stuff like that. And then just from the clinic side from being in quarantine and their numbers have gone up a lot on their sugars and blood pressure is not really well controlled anymore.”

“The coronavirus is touching too many people, and this is the topic of the day. But I feel that the basis in that global training where we cannot separate the vaccine from the diet, from the physical training, that is, it is a comprehensive training to manage a new lifestyle.”

### 3.2. Development and implementation of CHW/R workforce capacity building strategies

NAU-AzCHOW held regular virtual meetings throughout the research process, equitably contributing to the development of data collection tools, participant recruitment, and data analysis and interpretation – each team leveraging their respective and collective expertise, capacity, and strengths. While NAU researchers led data collection and analysis, information shared during focus groups was discussed in weekly team meetings with AzCHOW. Team meetings consisted of a trusted space where NAU and AzCHOW staff engaged in open knowledge sharing of salient themes from focus groups as well as anecdotes from personal relationships with CHW/Rs and community members. In line with AZ-CEAL aims 2 and 3, AzCHOW then led the development of health education materials and training for CHW/Rs to use with their communities based on priority topics identified from team conversations.

NAU-AzCHOW partnered to engage in CHW/R workforce capacity building during the pandemic, using focus group conversations to identify priority educational topics for CHW/Rs to better support their clients. As a result, AzCHOW developed and offered topic specific training in English and Spanish to the CHW/R workforce in Arizona, focusing on COVID-19 vaccines and spanning topics such as vaccine content, safety, and effectiveness, vaccine hesitancy, and addressing misinformation. Training style and delivery format were also driven by CHW/R input. For example, to address safety concerns and reach as many CHW/Rs as possible, synchronous training on vaccine topics and COVID-19 misinformation were offered virtually *via* Zoom during a time in the pandemic when vaccines were not widely accessible to all communities and vaccine uptake was low. Furthermore, AzCHOW adapted and expanded the synchronous virtual training on vaccines to an asynchronous online format, consisting of a 7-part series of training videos (35) on the following topics: (1) introduction to the COVID-19 vaccine video series; (2) COVID-19 vaccines available; (3) the development of COVID-19 vaccines; (4) deciding to get vaccinated; (5) speaking with clients about the vaccines; (6) COVID-19 vaccine myths or facts; and (7) educational resources. The 7-part series was created with intentionally short and concise videos, broken up by topics so that CHW/Rs could engage in learning at their own pace and be able to use the videos with their clients to provide education. The series was made available online on the NAU CEAL website in both English and Spanish and shared *via* the AzCHOW email listserv, including with CHW/Rs who participated in the focus groups.

Responding to CHW/R needs as expressed during focus group conversations, AzCHOW also developed and offered an online synchronous training focused on mental health support for CHW/Rs themselves, including topics of grief, isolation, and loss in the context of COVID-19. When vaccines became widely available to the public, AzCHOW developed and offered another online synchronous training for CHW/Rs on using motivational interviewing skills to build vaccine confidence in vaccine-hesitant clients. To date, AzCHOW has trained over 220 CHW/Rs representing 30 employers across the state of Arizona, including tribal CHR programs, federally qualified community health centers, and organizations offering social services, on priority topics identified during the focus group conversations. NAU and AzCHOW are also co-developing and implementing tools to evaluate and improve CHW/R training.

The NAU-AzCHOW team experienced some difficulties in developing the CHW/R training based on priorities identified during the focus groups. Evidence-based CHW/R curriculum and training to model in the design of training and other materials were limited. This required more creativity, and therefore additional time, from the team to develop training and materials. Another challenge was the ever-evolving nature of COVID-19 information, demanding that content be frequently updated. Similarly, information needed to be simplified into plain language for CHW/Rs to understand and be able to use with their clients. Training and materials then had to be translating into Spanish, ensuring cultural relevance in the translation. Translation resulted in time constraints since it could only be done by bilingual, bicultural team members. Furthermore, training and materials were pilot tested before full launch to ensure that content and delivery were effective and relevant. However, pilot testing was inconsistent, primarily due to staff time limitations, availability of CHW/Rs, and urgency (i.e., CHW/Rs needed up-to-date COVID-19 information quickly).

Despite challenges, NAU and AzCHOW effectively partnered to use research findings to inform rapid decision-making and action in the COVID-19 response. As COVID-19 continues to evolve, AZ-CEAL has also adapted to be culturally responsive and relevant to community needs. The latest effort of the NAU-AzCHOW partnership is a movement toward long COVID, with development of a training for CHW/Rs spanning topics of what long COVID is, prevention, management, and resources.

## 4. Discussion

During the pandemic, CHW/Rs have experienced a heightened role in supporting clients, including sharing critical and accurate information about COVID-19, linking them to essential services across the social determinants of health, and providing social connection during a time of isolation. Despite their documented effectiveness across health outcomes and settings on a global scale, CHW/Rs continue to be insufficiently engaged as experts in the US health care and public health systems, including in the pandemic response (11, 16, 17). The importance of CHW/Rs, including their role as key players in the frontline COVID-19 pandemic response, must be acknowledged with action that invests in a sustainable national CHW workforce. Building strong organizational culture supportive of CHW/R professional development and teams to respond to COVID-19 is essential.

### 4.1. CHW/R public health recommendations

CHW/R focus group participants raised a number of primary takeaways for an effective community-grounded pandemic response to address pressing needs in their Latinx and American Indian clients and support the prevention of COVID-19:

1. Implement culturally and linguistically relevant policies, programs, and resources to support clients at high risk for COVID-19, including non-English speaking, immigrant, elderly, homebound, and homeless communities.
2. Develop culturally and linguistically relevant mental health resources to support the isolation, grief, and loss disproportionately experienced due to COVID-19.
3. Create culturally and linguistically relevant materials to support COVID-19 prevention behaviors, including child and family member isolation due to schools' closure and quarantine.
4. Integrate COVID-19 prevention with chronic disease management, especially during periods of isolation and quarantine.
5. Improve public trust in public health recommendations and messaging to dispel myths and correct misinformation.
6. Invest in the professional development and preparation of the CHW/R workforce to serve their communities with culturally and linguistically relevant evidenced based materials and tools.

Using highly participatory methods, this academic-community partnership supported CHW/R workforce capacity building efforts in the context of COVID-19 to contribute to a community-grounded pandemic response. Our work aligns with best practices in CHW/R workforce engagement in research (28), contributing to better production of knowledge guided by CHW/R expertise. This is exemplified through a collaborative approach with the statewide association AzCHOW to develop data collection tools together, engage CHW/Rs throughout the research process such as in pilot testing the tools, and hold focus groups and special topic training in both English and Spanish.

Uniquely, NAU and AzCHOW teams reflect the diversity of communities of focus for AZ-CEAL, including bicultural, bilingual, racially-ethnically, and geographically diverse team members with a mix of professional and personal lived experiences. Throughout the research process, NAU-AzCHOW partners engaged in ongoing and open dialogue about what CHW/Rs on the ground were reporting as priority community concerns, needs, and challenges. CHW/Rs informed the development of culturally and linguistically relevant health education messages, materials, and training for CHW/Rs.

The CHW/R workforce in Arizona includes a unique population of Spanish-speaking CHWs or promotores de salud serving Latinx clients and indigenous tribally-employed CHRs serving AI clients. Unique to this study is the integration of perspectives of promotores and CHRs, which have historically distinct interests due to differences in origins and populations served among other factors (36). Building capacity across the two is essential in unifying and strengthening the CHW/R workforce, which in turn better positions the workforce for continued recognition and funding (36). The work of NAU-AzCHOW through AZ-CEAL provides a distinctive example of how research teams and statewide CHW associations can partner to facilitate knowledge sharing and create community-grounded resources to support CHW/R workforce capacity building in the context of a pandemic.

## 5. Conceptual or methodological constraints

Given the small sample size and focus on CHW/Rs employed in Arizona and serving primarily Hispanic/Latinx and American Indian clients, the research findings may not be generalizable to other communities or regions in the US. Another limitation of this study is the lack of African American CHW/R client representation. CEAL aims to work closely with the communities who have been most impacted by the pandemic, including Hispanic/Latinx, American Indian, and African American populations. Multiple attempts through a variety of networks to engage African American CHW/Rs in focus group conversations, including by research staff identifying as African American, were unsuccessful due to lack of strong relationships between the research team and organizational partners. This may also be explained partly by the low percentage of African American residents in the state compared to nationally (5.2 vs. 13.4%, respectively) (24). For the survey data, bivariate analyses were conducted and no significant differences were found between Latinx and AI participants. Additionally, since a majority of participants identified as women (89%), bivariate analysis in gender identity was not appropriate. Therefore, all results were reported aggregated.

## Data availability statement

The original contributions presented in the study are included in the article/supplementary material, further inquiries can be directed to the corresponding author.

## Ethics statement

The studies involving human participants were reviewed and approved by the University of Arizona Institutional Review Board (Protocol # 2011244240). Written informed consent for participation was not required for this study in accordance with the national legislation and the institutional requirements.

## Author contributions

DJ and SS lead the writing of the community case study. OG, RM, AP, LE, NL, MI, KG, RR, FR, HW, SO, and SP participated in the development of the research protocol, including its conceptualization and data collection tools. DJ, OG, RM, AP, LE, KG, RR, FR, and SS were involved in data collection, analysis, and interpretation. Each provided a detailed review of focus group data draft reports and reviewed the final versions of the community case study. All authors contributed to the article and approved the submitted version.
